# Detection of seed purity of hybrid wheat using reflectance and transmittance hyperspectral imaging technology

**DOI:** 10.3389/fpls.2022.1015891

**Published:** 2022-09-28

**Authors:** Han Zhang, Qiling Hou, Bin Luo, Keling Tu, Changping Zhao, Qun Sun

**Affiliations:** ^1^ Department of Seed Science & Biotechnology, The Innovation Center (Beijing) of Crop Seeds whole-process Technology Research Ministry of Agriculture and Rural Affairs (MOA), Beijing Key Laboratory of Crop Genetic Improvement, College of Agronomy and Biotechnology, China Agricultural University, Beijing, China; ^2^ Research Center of Intelligent Equipment, Beijing Academy of Agriculture and Forestry Sciences, Beijing, China; ^3^ Institute of Hybrid Wheat, Beijing Academy of Agriculture and Forestry Sciences, Beijing, China

**Keywords:** hybrid wheat, seed purity, hyperspectral imaging, reflectance spectrum, transmittance spectrum, machine learning

## Abstract

Chemical hybridization and genic male sterility systems are two main methods of hybrid wheat production; however, complete sterility of female wheat plants cannot be guaranteed owing to the influence of the growth stage and weather. Consequently, hybrid wheat seeds are inevitably mixed with few parent seeds, especially female seeds. Therefore, seed purity is a key factor in the popularization of hybrid wheat. However, traditional seed purity detection and variety identification methods are time-consuming, laborious, and destructive. Therefore, to establish a non-destructive classification method for hybrid and female parent seeds, three hybrid wheat varieties (Jingmai 9, Jingmai 11, and Jingmai 183) and their parent seeds were sampled. The transmittance and reflectance spectra of all seeds were collected *via* hyperspectral imaging technology, and a classification model was established using partial least squares-discriminant analysis (PLS-DA) combined with various preprocessing methods. The transmittance spectrum significantly improved the classification of hybrids and female parents compared to that obtained using reflectance spectrum. Specifically, using transmittance spectrum combined with a characteristic wavelength-screening algorithm, the Detrend-CARS-PLS-DA model was established, and the accuracy rates in the testing sets of Jingmai 9, Jingmai 11, and Jingmai 183 were 95.69%, 98.25%, and 97.25%, respectively. In conclusion, transmittance hyperspectral imaging combined with a machine learning algorithm can effectively distinguish female parent seeds from hybrid seeds. These results provide a reference for rapid seed purity detection in the hybrid production process. Owing to the non-destructive and rapid nature of hyperspectral imaging, the detection of hybrid wheat seed purity can be improved by online sorting in the future.

## Graphical Abstract

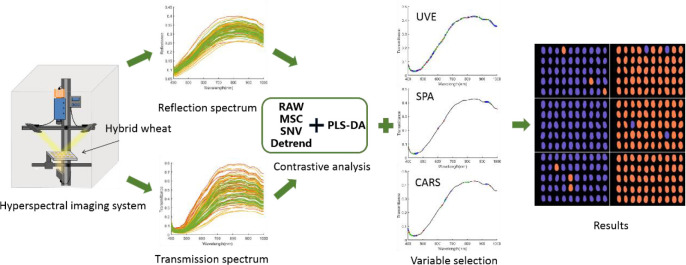

## 1 Introduction

Wheat is one of the top three global staple crops and contributes to approximately 20% of the global dietary energy (Singh et al., 2014; Boeven et al., 2016). The application of wheat heterosis can improve yield potential and stability, which is considered an important approach to overcome food shortage (Boeven et al., 2018). Currently, the chemical hybridizing agent (CHA) and genic male sterility (GMS) systems are widely used as a two-line hybrid system in hybrid wheat production because they do not require maintenance or any pre-propagation (Zhao, 2010; Murai et al., 2016; Li et al., 2020). However, CHA and GMS are highly dependent on the crop growth stage and weather, and the female parent is often not completely sterile (Singh et al., 2010). Even in certain promoted varieties, the fertility of the female parent under sterile conditions is still as high as 5%, which can affect the purity of hybrid seeds (Singh et al., 2021). Therefore, methods for accurate classification of hybrid seeds are required to ensure high seed purity.

Traditional methods for detecting the genuineness of seed varieties include seedling morphology detection, morphological detection, isozyme gel electrophoresis, and simple sequence repeat (SSR) analysis (Perry and Lee, 2015; Liu et al., 2022). These methods are limited by their destructiveness, complicated operation, high cost, and slow process, and thus cannot be used for rapid online detection in the seed processing industry (Tu et al., 2021).

Hyperspectral imaging (HSI) is a new detection technology that integrates spectroscopy and machine learning and can simultaneously obtain spectral and spatial information of samples. Owing to its non-destructive and rapid process, HSI has been widely used in food, medical, agricultural testing, and many other fields in recent years (Xing et al., 2019; Yoon et al., 2019; Feng et al., 2020; Wang et al., 2022). HSI can analyze the sample composition and characteristics at the molecular level (Yin et al., 2017). Spectral information obtained varies owing to phenotypic differences between seeds of different varieties. Therefore, researchers have used HSI techniques to detect and classify different seed varieties. Near-infrared HSI (NIR-HSI) has been used to identify different wheat populations (Choudhary et al., 2009). Visible-near-infrared (VIS/NIR) hyperspectroscopy has been used to identify wheat gluten (Zhu et al., 2012). In addition, hyperspectral detection technology has also been used to identify different varieties and contaminants of einkorn wheat (Ravikanth et al., 2016; Bao et al., 2019). Classification of seeds based on hyperspectral data has been widely performed, and reliable results have been obtained in the identification of seeds of different varieties. These studies have demonstrated the potential of HSI applications in seed variety classification.

The seed coat is developed from the ovary wall and integument of the female parent (Matzke and Riederer, 1990). Seeds of different varieties develop in different mother plants, and the composition of the seed coat varies. However, the hybrid is developed from the female parent and its seed coat composition is similar to that of the female parent seed. Some studies have shown that hybrids have a more consistent distribution of reflectance spectra with the female parent than that with the male parent or other seeds (Wiwart et al., 2014; Xu et al., 2017; Yang et al., 2018). Ran et al. (2017) used NIR-HSI to distinguish corn hybrids from female parents, with an average correct recognition rate of 85%, which is lower than that of traditional methods such as SSR analysis and isoenzyme gel electrophoresis. Therefore, the discrimination of hybrids and their female parent seeds is more difficult when compared to seeds of different varieties.

VIS/NIR transmittance spectroscopy is often used as a non-destructive testing method to evaluate food quality and detect internal damage in fruits (Dong et al., 2019; Huang et al., 2021). Compared to the reflectance spectra, transmittance spectra reflect deeper regions of the fruit (McGlone and Martinsen, 2004). Qin et al. (Qin et al., 2016) used reflectance and transmittance spectra to identify haploid corn kernels with an identification accuracy of 93.2% for the transmittance pattern which is considerably higher than <60% for the reflection pattern. In addition, the transmittance spectrum is superior to reflectance spectrum in the detection of seed mildew (Pearson et al., 2001; Lu and Ariana, 2013). These studies showed that transmittance spectroscopy should be preferred over reflectance spectroscopy in situations where it is necessary to detect differences in deeper regions of samples. The composition of the hybrid and the female parent on the seed coat is similar, but the internal composition of the seed is affected by the genes of both parents, which is different from the female parent. Therefore, the detection of deeper regions of the sample is more reflective of the differences between hybrids and their parent seeds.

To ensure the purity of hybrid wheat seeds, we aimed to identify and classify seeds and their parents by combining reflectance or transmittance VIS/NIR hyperspectral data with machine learning algorithms in this study. The specific research objectives are as follows: (1) detect the ability of VIS/NIR hyperspectral technology combined with machine learning algorithms to identify hybrids and their parental seeds; (2) analyze and compare the classification results of reflectance and transmittance hyperspectral data in classifying hybrids and their parental seeds; (3) select the best spectral preprocessing and feature extraction method, and establish the optimal wheat hybrid identification model against its female parent seeds; (4) explore the performance of the best detection model in the classification and recognition of seeds harvested in different years.

## 2 Materials and methods

### 2.1 Experimental samples

**Figure 1:**
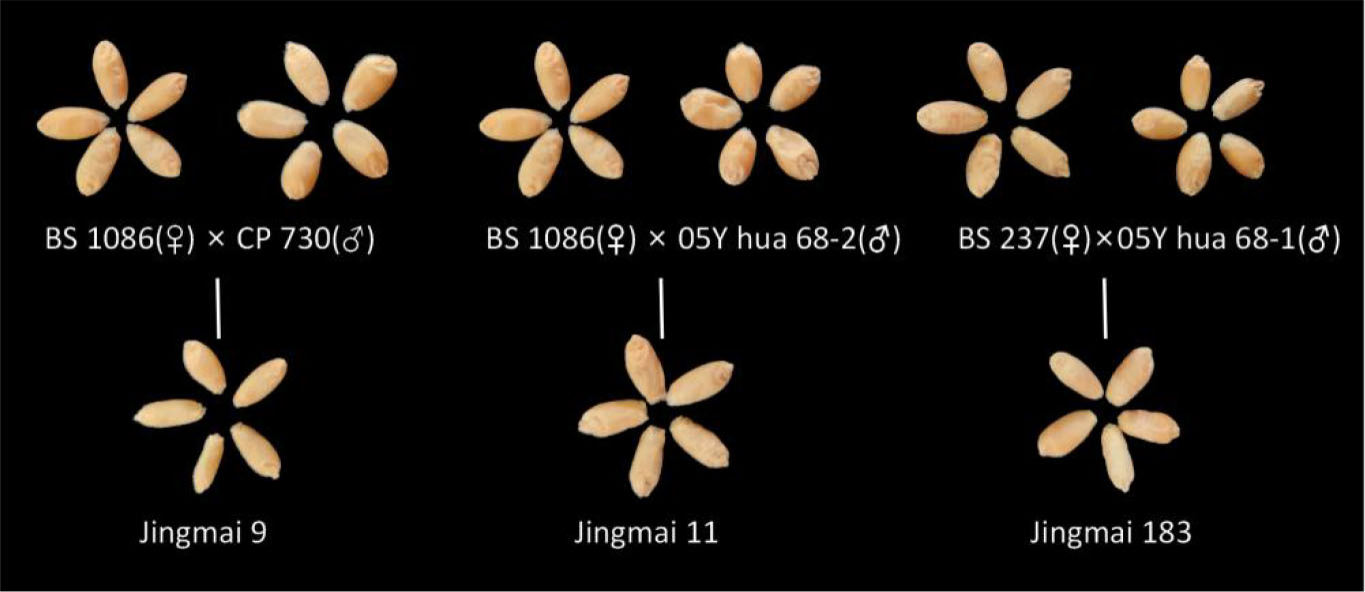
Images of wheat seeds and their pedigrees.

A total of eight wheat varieties were used in this study, including Jingmai 9, BS 1086, CP 730, Jingmai 11, 05Y hua 68-2, Jingmai 183, BS 237, and 05Y hua 68-1. Jingmai 9, Jingmai 11, and Jingmai 183 are hybrids, and others are parental seeds. Notably, Jingmai 9 and Jingmai 11 have a common female parent. These experimental samples were all high-purity original seeds provided by the Institute of Hybrid Wheat, Beijing Academy of Agriculture and Forestry, Beijing, China. Seeds were collected between 2020 and 2021, sealed in kraft paper bags, and stored in a dry environment. Before the experiment, withered or damaged wheat seeds were removed, and 204 seeds of each variety were randomly selected for data collection. The images of seeds of eight wheat cultivars and their pedigrees are shown in **Figure 1**. In addition, 50 seeds were randomly selected from every hybrid and their female parents produced in 2021, as well as Jingmai 9 and the female parents produced in 2020, to test the performance of the model. None of these seeds were involved in modeling and were solely used to verify the actual detection accuracy of the model.

### 2.2 HSI and spectral data collection

#### 2.2.1 HSI system

**Figure 2:**
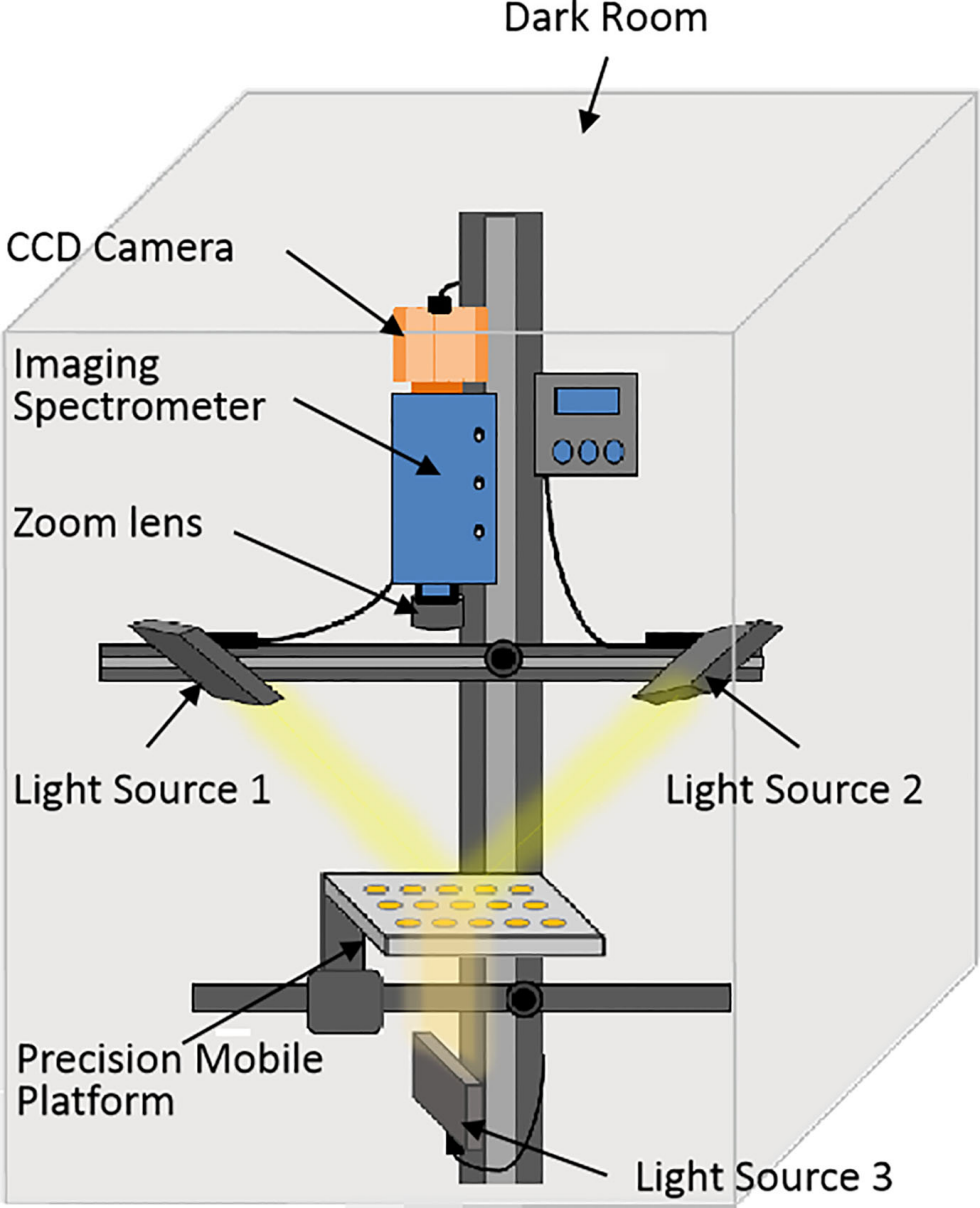
Hyperspectral imaging system.

The VIS/NIR HSI system used in the experiment mainly consisted of the following six components (**Figure 2**): a linear scanning V10E imaging spectrometer (Spectral Imaging Ltd., Oulu, Finland), a charge-coupled device camera (EM285CL; Raptor Photonics, Ltd., Larne, United Kingdom), zoom lens (OLE23; Schneider, Ratingen Germany), 150 W halogen tungsten lamp (IT 3900 e; Illumination Technologies Inc., New York, NY, USA), stepper motor-driven precision mobile platform (IRCP0076-1 COMB; Isuzu Optics Corp., Hsinchu, Taiwan), a computer equipped with Spectral Image software. The imaging spectral range of the system was 311–1,090 nm, the spectral resolution was 0.77 nm, and there were 1,002 bands in total. The No. 1 and No. 2 halogen lamps were located above the precision mobile platform, symmetrically distributed on both sides of the camera, and illuminated on the platform at 45° angle for reflectance spectrum collection. The No. 3 halogen lamp was located below the platform, in a vertical line with the camera, and illuminated from bottom to top for transmittance spectrum acquisition. All acquisitions are carried out in a dark room.

**Figure 3:**
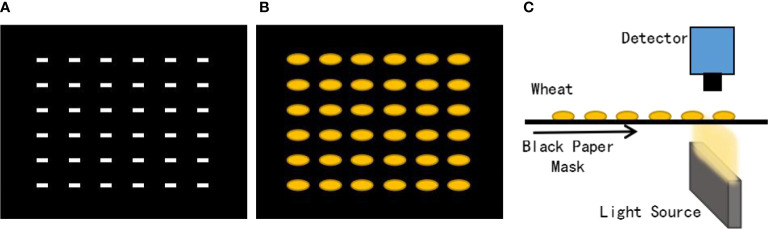
**(A)** A black paper mask (100 × 100 mm) containing small rectangular slits (2 × 4 mm) was used. **(B)** Wheat seeds were placed on the mask to avoid exposure to redundant light. **(C)** Schematic of transmittance hyperspectral image acquisition of wheat seeds.

To solely obtain the transmittance spectrum of seeds and reduce the influence of reflected light, a black cardboard mask (100 × 100 mm) containing tiny rectangular slits (2 × 4 mm) was prepared to hold the seeds (**Figure 3**). The seeds covered the slits entirely to reduce the influence of the reflected light generated by the surface of the light-scattering seed on transmittance spectrum acquisition when the spectral information was collected (Siedliska et al., 2017; Zhang et al., 2017).

A series of preliminary tests were performed before image acquisition to determine experimental parameters and system settings. Illumination power was determined by detecting the raw spectral intensity of wheat seed samples. Compared with reflection, light loses relatively more energy when penetrating the wheat sample. Therefore, it is necessary to adjust the light intensity by increasing the exposure time of the camera to ensure that the image intensity is at an appropriate level (<80% of the maximum pixel output of the camera) (Qin et al., 2016). A Teflon sheet was used for obtaining the white reference and is often used as a white reference for transmittance images in quality assessments of interior food regions (Leiva-Valenzuela et al., 2014; Hu et al., 2018). Further, we ensured that the image was not saturated when acquiring a white reference image by choosing a white reference plate of appropriate thickness. Finally, we determined that the thickness of the white reference plate was 4 mm, and the intensity at this time was close to 80% of the maximum pixel output of the camera.

When collecting the reflectance spectrum, the seeds were evenly placed on the black cardboard, the black cardboard was placed on the transfer table, and the No. 1 and No. 2 tungsten halogen lamps were turned on. When collecting the transmittance spectra, the hybrid seeds were evenly placed on a black cardboard mask containing rectangular slits, the black cardboard was placed on the transfer table, and the No. 3 tungsten halogen lamp was turned on.

To eliminate the influence of dark current and other noise on the image, the original hyperspectral image was corrected using the following formula:


(1) $$I_{c}=\frac{I_{raw}-I_{dark}}{I_{white}-I_{dark}}$$

Where *I_raw_* represents the raw hyperspectral image, *I_white_* represents the white image, and *I_dark_* represents the dark image. The dark reference image for the transmittance spectrum was acquired by completely covering the lens with an opaque cover. The white reference image was acquired by transmitting light through a white Teflon plate (4 mm thick) using light source 3. The dark reference images for the reflectance spectrum were acquired similar to the transmittance spectrum. In contrast, the white reference images were acquired using Teflon plates irradiated using light sources 1 and 2.

#### 2.2.2 Spectral collection

Owing to the instability of the spectral information in a single pixel, it is necessary to extract and calculate the average value of spectral information of all pixels in the same sub-projection area to obtain an average spectrum. After obtaining the hyperspectral image of seeds, threshold segmentation was used to remove the background spectral information to establish a mask, and the entire seed region of interest (ROI) was selected to extract the average spectrum, which represents a simple and efficient method and is widely used in hyperspectral image processing (Siedliska et al., 2017; Nie et al., 2019; Zhang et al., 2020). In this study, the reflectance and transmittance spectrum bands were at 450.2 nm and 865.2 nm, respectively, and the difference between the seed spectrum value and the background reached the maximum value. Therefore, we split the seeds in these two bands, built an ROI mask *via* threshold segmentation, and then extracted the average spectrum of each pixel in the ROI. To eliminate the influence of the external environment and camera performance, the front and back bands containing obvious noise were removed and 765 spectral bands in the range of 400–1,000 nm were obtained, which were used for discriminant analysis. The script for spectral extraction was written using Matlab2021b.

### 2.3 Data analysis

#### 2.3.1 Data preprocessing

Since the noise in the spectrum acquisition process interferes with subsequent data analysis, it is necessary to use an appropriate method to preprocess the original spectrum data to eliminate the background noise, baseline drift, stray light, and other interference signals during the spectrum acquisition process and to improve the model accuracy (Zhang et al., 2020). In this experiment, three methods, standard normal variable transformation (SNV), multiplicative scatter correction (MSC), and Detrend, were used to preprocess raw spectral data.

#### 2.3.2 Characteristic wavelength extraction

Hyperspectral data contains redundant feature variables and collinear adjacent bands, which can slow down modeling, affecting the speed and robustness of the model. Therefore, multivariate wavelength selection algorithms are usually used to obtain key wavelengths to establish simpler and improved quantitative models. In this study, uninformative variable elimination (UVE), successive projections algorithm (SPA), and competitive adaptive reweighted sampling (CARS) were used to extract characteristic wavelengths from the average spectrum of wheat seeds, respectively, to simplify the model and improve the reliability of the model (Cai et al., 2008; Song et al., 2016; Wang et al., 2021).

In this study, the components used to determine the criterion parameter in the UVE algorithm was set to 20 and the remaining parameters used default values in Matlab2021b (random variables: ‘pz’ = 200; cutoff level considered: ‘cutoff’ = 0.99). To improve the processing efficiency of the algorithm, the minimum and maximum number of variables in the SPA algorithm were set to 2 and 50, respectively. When CARS was used as the variable selection algorithm, the sampling number of Monte Carlo simulation was 500, the final variable number to be selected was determined *via* 5-fold cross validation, and the maximum number of latent variables for cross validation was 5.

#### 2.3.3 Partial least squares discriminant analysis (PLS-DA)

PLS-DA is a classification model widely used in chemistry, food science, and other fields (Rodionova and Pomerantsev, 2020). It is established based on PLS regression (PLSR) and its algorithm includes two key steps: PLSR fitting and class determination (Xia et al., 2019). This method combines the advantages of multiple linear regression and principal component analysis. PLS-DA can perform regression modeling under the conditions of many independent variables, multiple correlations, and poor correlation between independent variables (Wang et al., 2021).

During modeling, seeds of the three wheat hybrids were combined with their female or male parent seeds resulting in a total of 12 datasets. For each dataset, 80% of the samples were randomly selected as the training set to train a model and the remaining 20% of samples were used as testing set. The method was repeated 10 times for cross-validation to obtain the average classification accuracy. The RANDPERM operator in MATLAB was used for sample division.

## 3 Results and discussion

### 3.1 Spectral characteristics of hybrid wheat seeds

**Figure 4:**
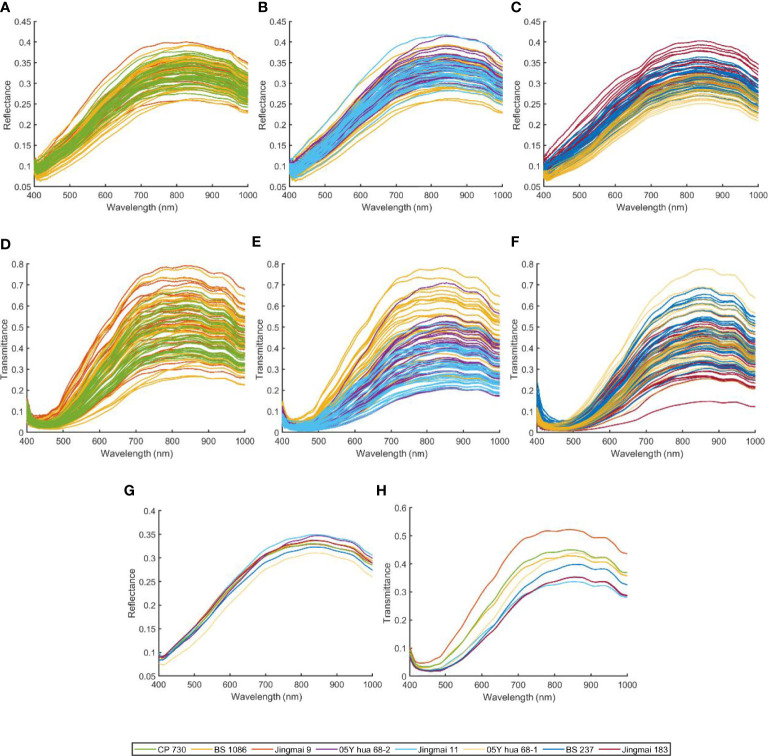
Spectral curve of hybrids and their individual parental seeds: raw reflectance spectra of **(A)** Jingmai 9, BS 1086, CP730, **(B)** Jingmai 11, BS 1086, 05Y hua 68-2, and **(C)** Jingmai 183, BS 237, 05Y hua 68-1; raw transmittance spectra of **(D)** Jingmai 9, BS 1086, CP730, **(E)** Jingmai 11, BS 1086, 05Y hua 68-2, and **(F)** Jingmai 183, BS 237, 05Y hua 68-1; **(G)** the average reflectance spectra of each wheat variety; **(H)** the average transmittance spectra of each wheat variety.

The spectral curves of seeds of the three groups of hybrid wheat and the parents are shown in **Figures 4A–F**, and the average spectra of each variety are shown in **Figures 4G, H**. The change in trends of reflectance and transmittance spectra of the three hybrid wheat seeds and the parental seeds was generally similar. However, the average reflectance and transmittance spectra of hybrid wheat seeds and their parents were not identical. This may be due to genetic differences caused by cross-breeding manifested as differences in gene expression levels (Nie et al., 2019). The reflectance spectra of the eight wheat species were relatively smooth and had no obvious spectral absorption peaks (**Figure 4G**), while three absorption peaks were detected in the spectrum of the average transmittance spectrum (the peaks and valleys were located at 450 nm, 900 nm, and 980 nm, respectively; **Figure 4H**). The band at approximately 450 nm is in the range of blue light, and certain specific bands are related to the pigments of plants, such as chlorophyll II a, chlorophyll II b, and carotenoids (Zhang et al., 2020). The band at 900 nm may be related to the third overtone of the C-H stretch. The spectral wavelength at 980 nm can be attributed to the O-H stretch second overtone (Cen and He, 2007). However, the spectral curves of the three groups of hybrid wheat and its parents have a high degree of overlap. It is unreliable to distinguish hybrid wheat from its parents only by the spectral curves’ difference in reflectance and transmittance values. However, the spectral curves of the three groups of hybrid wheat and its parents had a high degree of overlap. The discrimination of hybrid wheat from its parents based on the difference in spectral curves between reflectance and transmittance values alone is unreliable.

**Figure 5:**
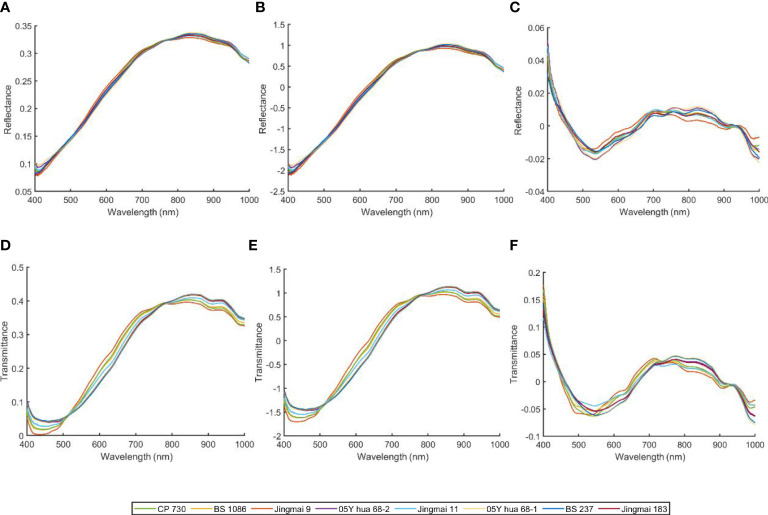
Spectral curves of different preprocessing methods: reflectance spectral curves after **(A)** MSC processing, **(B)** SNV processing, and **(C)** Detrend processing; transmittance spectral curves after **(D)** MSC processing, **(E)** SNV processing, and **(F)** Detrend processing. MSC, multiplicative scatter correction; SNV, standard normal variable transformation.

A high-frequency noise was detected in the original spectrum, indicating the need for preprocessing (**Figure 4**). Preprocessing improves the classification accuracy of the model in classifying wheat and its pollutants (Ravikanth et al., 2016). Therefore, the raw average spectral data obtained in this study were preprocessed using MSC, SNV, and Detrend (**Figure 5**). The relative differences between the average spectra of the three groups of hybrid wheat and their parents were reduced after MSC and SNV (**Figures 5A, B, D, E**). The spectral value range increased after SNV processing. The absorption peak increased remarkably after Detrend treatment (**Figures 5C, F**). However, neither the raw nor preprocessed mean spectra differed significantly among different seed varieties. Therefore, it is necessary to establish a discriminant model to identify and classify hybrid wheat and parental seeds.

### 3.2 Classification results and analysis of discriminant models based on full wavelengths

Based on the spectral data in the entire wavelength range for both transmitted and reflected light, the discriminant models of hybrid wheat mixed with female or male parent seeds were established by PLS-DA algorithm. The mean of the classification accuracy of 10 cross-validations of model were obtained, and the results are shown in **Table 1**. From the results of the PLS-DA model in **Table 1**, in the reflectance mode, the modeling results for identifying the hybrid and male parent seeds based on full-spectrum data are better than hybrid and female parent seeds. This had been in line with our research expectation that the embryo and endosperm of the hybrid seed contain both the genetic material of the male and the female parent, and show different phenotypes from their parents accordingly. However, the outer layers of wheat caryopses are composed of the pericarp, the actual seed coat, and remnants of nucellar tissues (Matzke and Riederer, 1990). These are developed from the ovary wall and integument, which leads to phenotypic similarity in seed coat between hybrids and their female parent. Therefore, the material composition of the seed coat of hybrid wheat seeds is similar to that of the female parent. This may also explain the higher accuracy in identification between hybrid and male parent seeds than that of hybrid and female parent seeds in reflectance mode.

**Table 1:** The classification results based on full spectrum using PLS-DA algorithm.

Group	Pretreatment	Jingmai 9 mixed with CP 730		Jingmai 9 mixed with BS 1086		Jingmai 11 mixed with 05Y hua 68-2		Jingmai 11 mixed with BS 1086		Jingmai 183 mixed with 05Y hua 68-1		Jingmai 183 mixed with BS 237	
		Tra/%	Tes/%	Tra/%	Tes/%	Tra/%	Tes/%	Tra/%	Tes/%	Tra/%	Tes/%	Tra/%	Tes/%
Reflectance	RAW	97.78	95.12	92.56	83.7	96.78	93.19	84.71	75.14	98.02	95.59	91.21	81.81
	MSC	99.30	98.41	95.71	88.76	98.11	95.01	89.99	80.27	98.23	95.26	96.55	89.15
	SNV	99.30	98.25	95.84	89.4	98.17	95.69	90.73	79.08	98.22	95.01	96.63	88.89
	Detrend	98.43	93.75	95.47	89.08	98.54	95.37	91.17	83.35	98.90	96.62	95.76	89.8
Transmittance	RAW	93.75	88.68	96.17	88.79	97.42	90.61	96.21	83.57	95.42	89.32	96.39	86.55
	MSC	94.48	89.41	98.05	92.06	97.24	94.73	97.4	91.36	92.67	86.28	95.24	88.70
	SNV	94.55	86.18	97.40	92.33	97.70	94.12	97.98	88.49	92.66	86.36	95.23	88.20
	Detrend	93.89	89.31	97.12	92.84	98.23	95.36	97.06	91.52	98.92	95.62	97.71	92.38

Tra, Classification accuracy of training set; Tes, Classification accuracy of testing set; RAW, raw spectral data; MSC, multiplicative scatter correction; SNV, standard normal variable transformation.

The accuracy of classification and identification of hybrid and female parent seeds based on modeling full-spectral data was improved in transmittance mode compared to that obtained in reflectance spectroscopy. Conversely, the classification accuracy of the hybrid and male parent seeds models decreased. Owing to the poor penetration of reflected light, diffuse reflectance spectroscopy can only obtain information on the surface of the grain. In contrast, the transmittance spectrum can enable the full accumulation of the depth information of the analytical optical path and information inside the sample (Qin et al., 2016). The difference between hybrid and female parent seeds is mainly reflected in the embryo and endosperm inside the seed; thus, transmittance spectrum modeling of the hybrid and female parent is more reliable than modeling reflectance spectrum. However, for hybrids and male parents with differences in the seed coat, the model established based on transmittance spectrum is not better than that using reflectance spectrum. Moreover, there was a significant decline in the classification accuracy of Jingmai 9 and the male parent. For the three groups of seeds used in this study, the transmittance spectrum classification results of hybrids and female parents were better than those obtained using reflectance spectrum classification. Among them, the transmittance spectrum classification effect after Detrend preprocessing was the best, and the classification accuracy of the testing set of Jingmai 9, Jingmai 11, and Jingmai 183 reached 92.84%, 91.52%, and 92.38%, respectively. Therefore, further characteristic wavelength screening was performed using the transmittance spectrum preprocessed by Detrend, and an analysis model was established to provide more references for the development of multi-spectral rapid detection systems.

### 3.3 Modeling analysis based on characteristic wavelengths

#### 3.3.1 Optimal wavelengths selection

**Figure 6:**
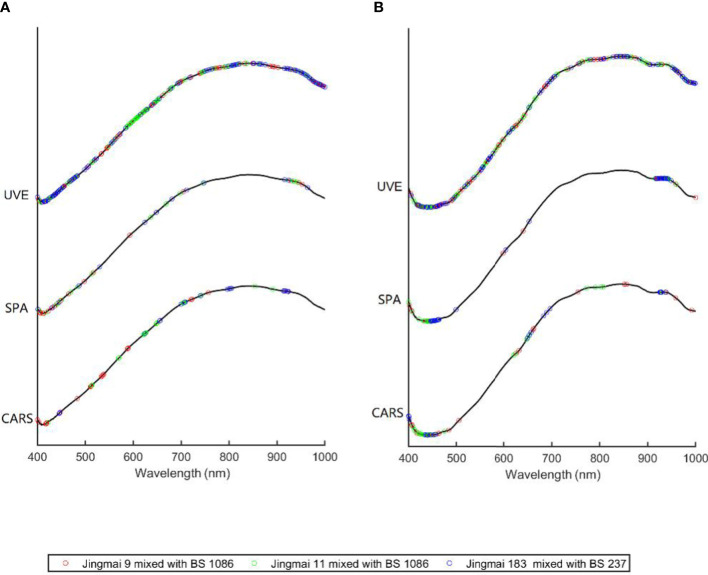
Detailed location of characteristic wavelengths screened using different methods: **(A)** reflectance spectrum and **(B)** transmittance spectrum.

The purpose of characteristic wavelength selection is to reduce the dimension of original high-dimensional spectral data, retain helpful information to the greatest extent, and eliminate redundant information. In this study, UVE, SPA, and CARS algorithms were used to re-model the selected characteristic bands from the complete spectral characteristics of the three hybrids and their female parent seeds after Detrend preprocessing. The specific wavelengths identified by the three variable selection algorithms are shown in **Figure 6**.

When UVE was used to select characteristic wavelengths in the transmittance spectrum, the number of spectral characteristic variables corresponding to different kinds of hybrid wheat was reduced from 753 to approximately 65–89. When SPA and CARS were used to select characteristic wavelengths, the number of variables was reduced from 753 to approximately 13–28. Few differences in the characteristic bands were screened out by the three characteristic wavelength algorithms in the transmittance and reflectance spectra (**Figure 6**). Compared with the reflectance spectrum, the characteristic wavelengths selected by different feature-screening methods were more concentrated in the transmittance spectrum. They were mainly concentrated in certain bands near 400–500, 630–650, and 910–1,000 nm. Few researchers have proposed that specific-wavelength bands in the visible light region may be related to plant pigments, such as chlorophyll II a at 430 nm, chlorophyll II b at 448 nm, carotenoids at approximately 448 nm and 471 nm, and anthocyanin at 623 nm, 642 nm, and 646 nm with absorption peaks (Sun et al., 2016; Zhang et al., 2020). Additionally, the spectral band in the range of 400–500 nm is related to the starch content of seeds, and the band at approximately 900–1,000 nm is considered to reflect the difference in seed protein content (Wang et al., 2022). The genes of the paternal parent influence the endosperm of the hybrid, and the starch and protein content of the hybrid showed few differences from the maternal parent, which were reflected in the corresponding bands of the spectrum. To further determine the optimal feature selection algorithm, further modeling analysis was performed based on the extracted characteristic wavelengths and the optimal feature selection algorithm was selected.

#### 3.3.2 Classification results and analysis based on characteristic wavelengths

The spectral data preprocessed using Detrend were subjected to dimensionality reduction transformations of UVE, SPA, and CARS, and then the hybrid and female parent seed classification models were established using PLS-DA (**Table 2**). In the feature band screening method, the accuracy rate of the model established *via* SPA and UVE processing decreased in certain varieties compared with that of the whole band. The accuracies of models established using CARS processing were improved compared with that of full-band modeling. The overall performance of the classification and identification of hybrids and female parents based on transmittance spectroscopy was still better than that of reflectance spectroscopy. Based on the results presented in section 3.3.1, the characteristic bands finally screened using CARS were below 30, which can effectively eliminate unusable spectral information, and the number of extracted bands was <3.2% of the full band, which was considerably lower than that obtained using UVE. Therefore, the Detrend-CARS-PLS-DA model based on the transmittance spectra was the best model for classifying wheat hybrid and female parent seeds. Finally, the classification accuracies of the established model in the testing sets of Jingmai 9, Jingmai 11, and Jingmai 183 were 95.69%, 98.25%, and 97.25%, respectively.

**Table 2:** The classification results of hybrids and female parent seeds based on different characteristics selection spectra.

Group	Wavelength selection	Jingmai 9 mixed with BS 1086		Jingmai 11 mixed with BS 1086		Jingmai 183 mixed with BS 237	
		Tes/%	No.	Tes/%	No.	Tes/%	No.
Reflected	UVE	90.13	81	84.67	86	89.43	89
	SPA	85.33	22	80.39	16	88.03	13
	CARS	92.65	23	91.44	18	92.94	18
Transmitted	UVE	92.35	80	94.12	65	93.01	79
	SPA	91.37	28	92.45	30	91.47	25
	CARS	95.69	26	98.25	28	97.25	20

Tes, Classification accuracy of testing set; No., Number of selected feature wavelengths; UVE, uninformative variable elimination; SPA, successive projections algorithm; CARS, competitive adaptive reweighted sampling.

#### 3.3.3 Optimal model validation and visualization

**Figure 7:**
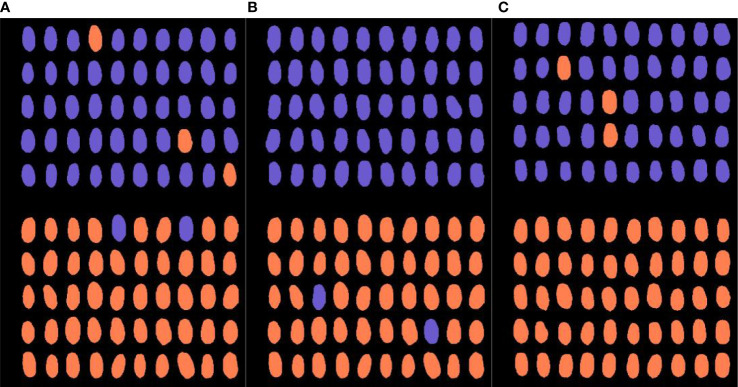
Visualization of hybrid seed versus female parent seed classification results: **(A)** Jingmai 9, **(B)** Jingmai 11, and **(C)** Jingmai 183.

In addition to the modeled sample of 1,632 seeds (204 seeds per category), this study selected 250 seeds (50 seeds per category for hybrid and female parent seeds) for optimal model validation and visualization. A visualization of the verification results is shown in **Figure 7**. Five, two, and three seeds were misidentified among Jingmai 9, Jingmai 11, and Jingmai 183, respectively (**Figure 7**); the validation accuracies of the three hybrids were 95%, 98%, and 97%, respectively; which was consistent with the modeling validation set (**Table 2**). Therefore, the model can maintain stable accuracy when detecting the same batch of seeds. This method can quickly perform preliminary detection of hybrid seed purity to identify samples with a high contamination ratio. Additionally, owing to the non-destructive detection characteristics of HSI, this method can be used for the online selection of hybrid seeds, and the purity of hybrid wheat samples can be improved by separating female parent seeds. Considering Jingmai 9 as an example, for Jingmai 9 seeds with a purity of 90%, the seeds identified as the female parent were filtered out *via* the aforementioned method which increased the seed purity to approximately 99%.

### 3.4 Validation of the detection model for the seed of different years

When hyperspectral data are obtained, their analysis is limited by modeling samples. While modeling can often maintain a high accuracy rate when detecting samples of the same batch in the same year, the accuracy significantly decreases when testing across different years or seed lots (Guo et al., 2017). Therefore, we selected the hyperspectral transmittance images of 50 seeds of Jingmai 9 and BS 1086 wheat seeds harvested in 2020 to verify the detection accuracy of the best purity detection model for seeds across years and visualized the results.

**Figure 8:**
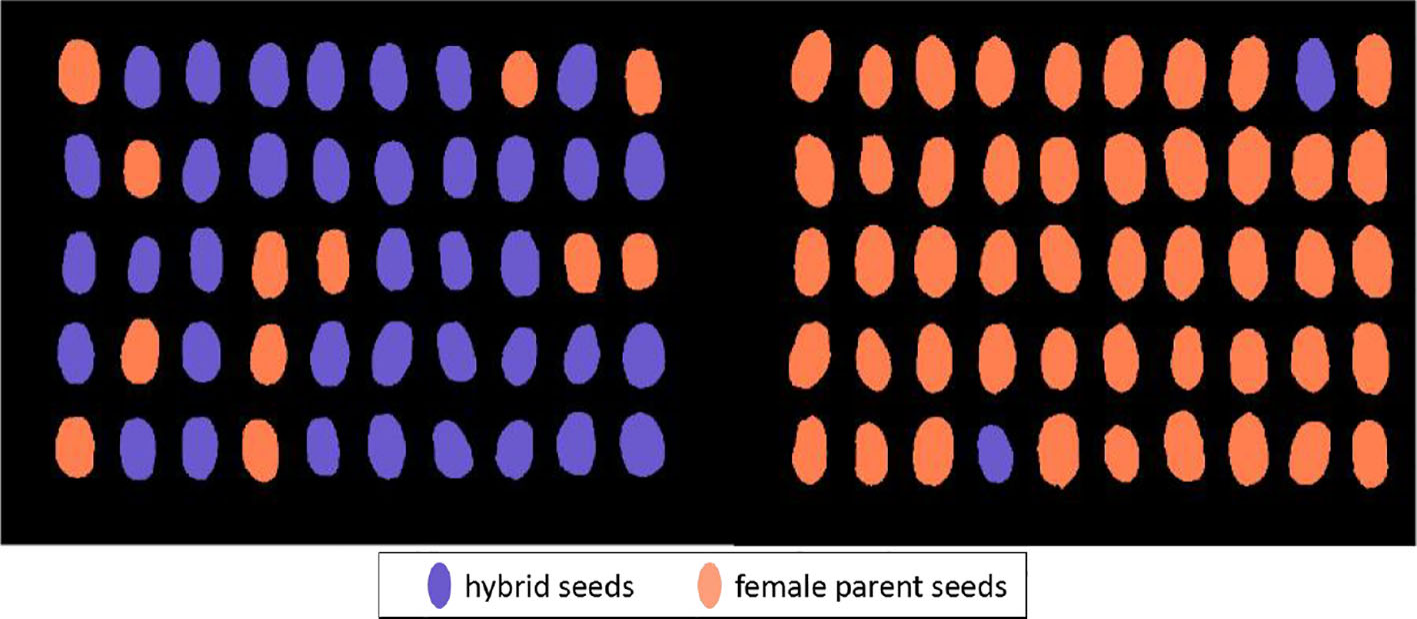
Visualization of detection of seeds of Jingmai 9 and its female parent.

The cross-year detection results of Jingmai 9 and the female parent are shown in **Figure 8**. The final detection accuracy was 86%. Few identification errors were expected and since the training samples in the model did not contain seeds harvested in 2020 and seeds of the same variety harvested in different years have phenotypic differences. Compared with the seed samples harvested in 2021, the model classification accuracy significantly decreased based on the confounding of Jingmai 9 and female parent seeds harvested in 2020. Moreover, in the cross-year prediction of the model established for Jingmai 9 seeds mixed with its female parent seeds, only two female parent seeds were incorrectly identified as Jingmai 9, and 12 seeds of Jingmai 9 were predicted as the female parent seeds. Since the proportion of female parent seeds in hybrid wheat will be relatively small in actual production, the accuracy of this model used in assessment of actual seed purity will be further reduced. However, for seed sorting, owing to the high recognition precision of female parent seeds, this model can accurately discriminate female parent seeds from the hybrid sample. In this study, the purity detection model for hybrids against their female parents established using transmitted light can achieve good results in detecting seeds from the same lot. However, the detection accuracy may decline when analyzing seeds from different years. In further studies, it will be necessary to add standard samples from different years and growing environments to improve the prediction accuracy of the model.

### 3.5 Analysis of modeling results

In this study, we used transmittance and reflectance HSI to classify hybrids and their parent seeds, respectively. Reflectance HSI was more efficient in identifying and classifying hybrid and male parent seeds than when used for classifying hybrid and female parent seeds (**Table 1**). Hybrid and male seeds are harvested from plants of different varieties, and there are differences in the seed coat. Therefore, we obtained good results in identifying hybrid and male seeds using reflectance HSI, which can effectively identify differences in seed coats.

The influence of the maternal parent, including maternal cytoplasmic inheritance, genomic imprinting, and maternal effect (Wolf and Wade, 2009), on the formation of offspring is greater than that of the paternal parent. In Jingmai 11 and Jingmai 183, the optimal identification accuracies of the testing set of the hybrid and the male parent in the transmittance spectrum were 95.36% and 95.62%, respectively, which were better than the accuracies of 91.52% and 92.83% for the hybrid and the female parent, respectively (**Table 1**).

The transmittance spectrum is considered to enable the full accumulation of the optical path depth information to obtain information inside the sample (Qin et al., 2016). In our study, transmittance HSI was better than reflectance HSI in classifying and identifying the three hybrids and female parent. For the accumulation of internal depth information, the transmittance spectrum can reflect certain compositional differences inside the seeds. Therefore, transmittance hyperspectroscopy is considered suitable for the classification and identification of hybrids and female parents.

Transmittance spectra of single seeds can be considered a worst case with large additive and multiplicative scatter effects due to differences in kernel size, structure, and presentation angle (Pedersen et al., 2002). This results in a large variance in the transmittance spectrum measurements due to uncontrolled changes in light scattering (**Figure 4**). Although we have processed the spectra using a preprocessing method, the transmittance spectra still do not perform as well as the reflectance spectra in the identification of hybrids that are already distinct from the male parent in the seed coat. Among them, the optimal recognition effect for Jingmai 9 and male parent in transmittance spectrum was only 89.41%.

This study distinguishes hybrids from females in the 400–1,000 nm band based on variables selected *via* three feature-screening algorithms. The characteristic wavelengths selected for the transmittance spectrum are concentrated in specific wavelength bands such as 400–500 nm, 630–650 nm, and 910–1,000 nm. These bands of 400–500 nm and 630–650 nm correspond to the phytochromes of seeds (Sun et al., 2016; Zhang et al., 2020), which may be related to wheat seed coats. It is related to the precipitation of pigment content in the aleurone layer. The 910-1000nm band corresponds to the protein content of seeds (Wang et al., 2022), which reflects the difference in components between the hybrid and the female parent due to the genetic influence of the two parents. After feature screening, the model can still maintain a high prediction accuracy, reduce the number of spectra, and provide a reference for future multispectral detection.

In addition, the accuracy of the model decreased in the detection of Jingmai 9 and female parent seeds across years (**Figure 8**). The growth environment of seeds may differ across years, such as: rainfall, temperature, soil fertility. In practical applications, multi-year seeds should be added for modeling to further improve the transferability of the model.

At present, the actual production of wheat hybrids mainly involves the self-crossing of the female parent to produce the female parent’s seed-contaminated hybrid. Our results show that compared with reflectance HSI, transmittance HSI can more accurately identify hybrid and female parent seeds. It can provide a reference for pure sampling detection and online sorting of hybrids.

## 4 Conclusion

In this study, seeds of three pairs of different wheat hybrids and their parents were identified and classified using HSI technology combined with a PLS-DA model. The reflectance and transmittance modes were used for comparison and analysis, respectively. Combined with different preprocessing methods and feature extraction algorithms, a fast classification and identification method was established for discriminating hybrids and their female parent seeds. The established model can efficiently and non-destructively differentiate seeds from hybrids and their female parent seeds. The following specific conclusions can be drawn from this study:

1) Based on the similar seed coat phenotype, the identification accuracy of hybrid wheat and the female parent seeds was lower than that of hybrid wheat and male parent seeds when using reflectance spectrum for modeling and classification. The three wheat hybrids and their female parent seeds selected in this study were modeled in the hyperspectral full-spectral reflectance mode, and the recognition and classification accuracies were <90%.

2) The established transmittance hyperspectral model performed better than the reflectance model in classifying three hybrids and female parent seeds. The transmittance spectrum significantly improved the hybrid classification effect of hybrids and female parents compared to that obtained using reflectance spectrum.

3) After multivariate data analysis, the Detrend-CARS-PLS-DA model established using transmittance HSI showed best performance in classifying and identifying hybrid wheat and their female parent seeds. The classification and recognition accuracy of the testing set of Jingmai 9, Jingmai 11, and Jingmai 183 hybrids reached 95.69%, 98.25%, and 97.51%, respectively.

This method established by using transmittance spectroscopy combined with machine learning can accurately identify hybrid and female parent seeds. It can be widely used in the supervision and detection of wheat hybrid production and also in the timely detection of hybrid seed lots of low purity. In addition, combined with HSI-based non-destructive and rapid detection characteristics, this study provides a reference for the development of hybrid seed online detection and sorting systems in the future.

## Data availability statement

The raw data supporting the conclusions of this article will be made available by the authors, without undue reservation.

## Author contributions

HZ: Conceptualization, Methodology, Investigation, Writing - original draft. QH: Methodology, Investigation. BL: Formal analysis. KT: Writing - review & editing. CZ: Conceptualization, Methodology. QS: Conceptualization, Methodology. All authors approved the final version.

## Funding

This work was supported by the Beijing Academy of Agriculture and Forestry Sciences [grant numbers QNJJ202104].

## Conflict of interest

The authors declare that the research was conducted in the absence of any commercial or financial relationships that could be construed as a potential conflict of interest.

## Publisher’s note

All claims expressed in this article are solely those of the authors and do not necessarily represent those of their affiliated organizations, or those of the publisher, the editors and the reviewers. Any product that may be evaluated in this article, or claim that may be made by its manufacturer, is not guaranteed or endorsed by the publisher.
